# Identification of a Receptor for Neuropeptide VGF and Its Role in Neuropathic Pain*

**DOI:** 10.1074/jbc.M113.510917

**Published:** 2013-10-08

**Authors:** Ya-Chun Chen, Alessandro Pristerá, Mahmood Ayub, Richard S. Swanwick, Kersti Karu, Yosuke Hamada, Andrew S. C. Rice, Kenji Okuse

**Affiliations:** From the ‡ Department of Life Sciences, Faculty of Natural Sciences, Imperial College London, London SW7 2AZ, United Kingdom and; the § Department of Surgery and Cancer, Faculty of Medicine, Imperial College London, London SW10 9NH, United Kingdom

**Keywords:** Calcium Intracellular Release, Macrophages, Neurons, Neuropeptide, Pain, VGF, gC1qR, Neuropathic Pain, Sensory Neurons

## Abstract

VGF (nonacronymic) is a neuropeptide precursor that plays multiple roles in regulation of energy balance, reproduction, hippocampal synaptic plasticity, and pain. Data from a number of pain models showed significant up-regulation of VGF in sensory neurons. TLQP-21, one of the VGF-derived neuropeptides, has been shown to induce a hyperalgesic response when injected subcutaneously into the hind paw of mice. However, the precise role of VGF-derived neuropeptides in neuropathic pain and the molecular identity of the receptor for VGF-derived peptides are yet to be investigated. Here we identified gC1qR, the globular heads of the C1q receptor, as the receptor for TLQP-21 using chemical cross-linking combined with mass spectrometry analysis. TLQP-21 caused an increase in intracellular Ca^2+^ levels in rat macrophages and microglia. Inoculation of TLQP-21-stimulated macrophages into rat hind paw caused mechanical hypersensitivity. The increase in intracellular Ca^2+^ levels in macrophages was attenuated by either siRNA or neutralizing antibodies against gC1qR. Furthermore, application of the gC1qR-neutralizing antibody to rats with partial sciatic nerve ligation resulted in a delayed onset of nerve injury-associated mechanical hypersensitivity. These results indicate that gC1qR is the receptor for TLQP-21 and plays an important role in chronic pain through activation of macrophages. Because direct association between TLQP-21 and gC1qR is required for activation of macrophages and causes hypersensitivity, disrupting this interaction may be a useful new approach to develop novel analgesics.

## Teaser

**Background:** VGF is a neuropeptide involved in chronic pain.

**Results:** VGF-derived peptide TLQP-21 activates macrophages. We identified gC1qR as a receptor for TLQP-21.

**Conclusion:** TLQP-21 and gC1qR are involved in chronic pain pathways.

**Significance:** TLQP-21 and gC1qR may be drug targets for chronic pain treatment.

## Introduction

Peripheral neuropathic pain is a subtype of chronic pain caused by a variety of traumatic, chemical, and infectious insults to the peripheral nervous system. Despite considerable drug development investment, current treatments for neuropathic pain lack adequate efficacy and have unfavorable adverse effect profiles. It has been difficult to identify targets that were truly related to neuropathic pain as opposed to those that are merely epiphenomena related to nerve injury.

Recently, we compared primary sensory neuronal gene expression profiles in three disparate clinically relevant models of neuropathic pain (peripheral nerve trauma, HIV-associated neuropathy, and varicella zoster infection). We found VGF as a commonly up-regulated gene (1). VGF has also been found to be up-regulated in a number of neuropathic and inflammatory pain models (2–6). VGF was originally identified in PC12 cells as one of the proteins induced by nerve growth factor (NGF). VGF encodes a neuropeptide precursor with a restricted pattern of expression in neurons in the central/peripheral nervous systems. In the somatosensory system, small diameter neurons of dorsal root ganglia (DRG)^4^ and fibers in the superficial spinal dorsal horn express VGF. VGF undergoes endoproteolytic cleavage, and the products are released upon depolarization. Currently, several VGF-derived peptides have been identified, which are named by the first 4 amino acids and their overall length (*e.g.* TLQP-21). VGF plays multiple roles in regulation of energy balance, reproduction, and hippocampal synaptic plasticity. Functional roles of VGF-derived peptides in pain pathways have also been identified. Intrathecal application of TLQP-62, one of the longest VGF-derived peptides, to rats results in a long lasting mechanical and cold behavioral hypersensitivity (5). Injection of the shorter peptide TLQP-21 into the hind paw of mice resulted in hypersensitivity in both control animals and the formalin model of inflammatory pain (7). Intrathecal application of antibody against TLQP-21 attenuates the development of spared nerve injury-induced mechanical hypersensitivity (8). Another VGF-derived peptide, LQEQ-19, induced p38 MAP kinase phosphorylation in spinal microglia (6). These observations suggest that VGF-derived peptides have pronociceptive and hyperalgesic functions. Although VGF-derived peptides play important roles in pain modulation and many other functions, receptors for the peptides have yet to be identified. Recently TLQP-21 has been shown to bind to adipocyte membranes in a saturable manner (9), and atomic force microscopy of living cells revealed the existence of a single class of binding sites for TLQP-21 (10). These observations suggest the existence of a cell surface receptor for TLQP-21.

Here we identified gC1qR as the receptor for TLQP-21. TLQP-21 activates rat macrophages through gC1qR, and activated macrophages caused mechanical hypersensitivity in rats.

## EXPERIMENTAL PROCEDURES

### 

#### 

##### Intracellular Calcium Imaging

Rat primary microglia, macrophages, and DRG neurons were cultured as described (11–13). Cells were replated 24 h before imaging and kept in serum-free DMEM. Cells were incubated with 4 μm Fluo-4 AM (Molecular Probes, Invitrogen) for 30 min. Following three washes in extracellular solution (140 mm NaCl, 5 mm KCl, 1.8 mm CaCl_2_, 2 mm MgCl_2_, 10 mm d-glucose, 10 mm HEPES, pH 7.4), the cells were left for 30 min for de-esterfication. After three washes, cells were analyzed on a Leica SP5 confocal microscope. The Fluo-4 was excited with an argon laser at 488 nm, set at 10% of the maximum power, and emitted fluorescence was detected in the 500–570-nm range. Gain and offset of the photomultipliers were adjusted on the Leica LAS software. The recordings were taken every 2.6–6 s, and the pinhole aperture was set at the maximum value (9.89 Airy units or 600 μm, minimal confocality). For the Ca^2+^-free conditions, the CaCl_2_ in the extracellular solution was replaced with 2 mm EGTA and left for a further 10 min after the final washes. The peptides were synthesized by Peptide Protein Research, with >95% purity. The sequence of ScrTLQP-21 is PSFLLPPHHSRAQHRTPRAAR. Antibodies against gC1qR were from Abcam (MAb1, 60.11; MAb2, 74.5.2). siRNAs (3 μg) were transfected in 1 × 10^6^ macrophages in a volume of 50 μl by electroporation using a gene pulser II (Bio-Rad) at 300 V. The siRNA sequences against gC1qR are as follows: siRNA1, UAGGUGGUCAUACAAGGCCCA; siRNA2, UUCUCCGGCAACUUUGCGCAA; siRNA3, UAAUUUAGCCUCCGUGCCGTT; and siRNA4, UAAAUGGAGGUGUAACGGCGA.

##### Brain Homogenate and Monomeric Avidin Column Purification

200 mg of postnatal day 4 rat forebrain was homogenized in PBS with 20% glycerol, 0.1% Triton X-100, and mammalian protease inhibitor mixture (Sigma). The sample was centrifuged at 1000 × *g* for 10 min at 4 °C, and the supernatant was further centrifuged for 30 min at 100,000 × *g*. The pellet was resuspended in PBS/glycerol with 4% *n*-dodecyl-β-d-maltoside. The mixture was centrifuged for 30 min at 100,000 × *g*. The supernatant represented the solubilized membrane proteins. 10 μm biotinylated TLQP-21 in PBS with 20% glycerol, 0.1% *n*-dodecyl-β-d-maltoside, and 1 mm tris(2-carboxyethyl)phosphine was applied to the monomeric avidin-agarose column (Thermo Scientific) and left for 30 min to allow the peptide to attach completely. The membrane proteins were added to the column and eluted with PBS with 2 mm d-biotin.

##### Mass Spectrometry

Samples eluted from the column were resolved on SDS-PAGE. The gels were silver stained with SilverQuest (Invitrogen). The protein band at ∼30 kDa was cut, destained, and washed with 50 mm ammonium bicarbonate in 50% acetonitrile, reduced with 10 mm DTT and alkylated with 55 mm iodoacetamide and digested with 0.5 μg of trypsin (Promega) at 37 °C for 16 h. Peptides were extracted with 0.1% formic acid in 50% acetonitrile, lyophilized, and desalted in-house manufactured C_18_ purification tips. LC-MS/MS were performed on an Ultimate 3000 nano-HPLC system (Dionex) coupled to a LTQ XL Orbitrap mass spectrometer (Thermo Electron) operated in collision dissociation mode. Separation of peptides was achieved by reverse-phase chromatography on in-house packed Picotip emitter (New Objective) with ProntoSIL C_18_ phase (Bischoff Chromatography). MS data were acquired using a data-dependent acquisition mode and operated at 60,000 resolution (full width at half-maximum height, FWHM definition), and the top five 2+, 3+, and 4+ ions in the 300–1800 *m*/*z* were selected for MS/MS. Monoisotopic precursor selection was enabled, and fragmentation and dynamic exclusion with 40 s were enabled. Raw data files were searched against the NCBInr database using MASCOT with peptide mass tolerance ±20 ppm and fragment mass tolerance ±0.02 Da.

##### Quantitative Real-time RT-PCR and Western Blotting

Quantitative RT-PCR and Western blotting were performed as described (14, 15). RNA was extracted from the cells using an RNeasy kit (Qiagen) and reverse-transcribed by Superscript III (Invitrogen). Quantitative RT-PCR was performed on a StepOnePlus thermal cycler (Applied Biosystems), utilizing Fast SYBR Green Master Mix (Applied Biosystems). The housekeeping gene cyclophilin A was also amplified and used to normalize the amount of cDNA product for VGF. The threshold amplification cycles (Ct) were determined using StepOnePlus software and analyzed by the 2^−ΔΔCt^ method. The primers used for amplifying VGF and cyclophilin A mRNA were as follows: VGF forward, ACTTCCTGGTCCCAAACGG; VGF reverse, GGCTGGGAGACAGACACTTCA; cyclophilin A forward, TATCTGCACTGCCAAGACTGAGTG; cyclophilin A reverse, CTTCTTGCTGGTCTTGCCATTCC.

##### In Vivo Experiments

All animal experiments conformed to the British Home Office Regulations (PPL70/7162) and International Association for the Study of Pain guidelines for the care and use of animals. 3-week-old male Wistar rats were used for all experiments. Surgery was performed under general anesthesia with 1.0–2.0% isoflurane and 50% nitrous oxide in oxygen. Intraplantar injection of macrophages was performed by injecting 35,000 macrophage cells in PBS subcutaneously to the left hind paw of rats using a 30-gauge needle attached to a Hamilton syringe. For PSNL model, surgery was performed on the left sciatic nerve using 7.0 nonabsorbable silk sutures (Mersilk, Ethicon). The sciatic nerve was then wrapped loosely with an oxidized regenerated cellulose (16) strip previously soaked in PBS containing 25 μg of anti-gC1qR or control antibody. Mechanical sensitivity was examined by an electronic Von Frey test. The experimenter was blinded to the treatments received and had no knowledge of the experimental group to which an animal was randomized. Experiments were repeated three times, and three to five rats were examined in each experiment.

## RESULTS

### 

#### 

##### TLQP-21 Elicits Intracellular Ca^2+^ Increase in Macrophages and Microglia

**FIGURE 1.**
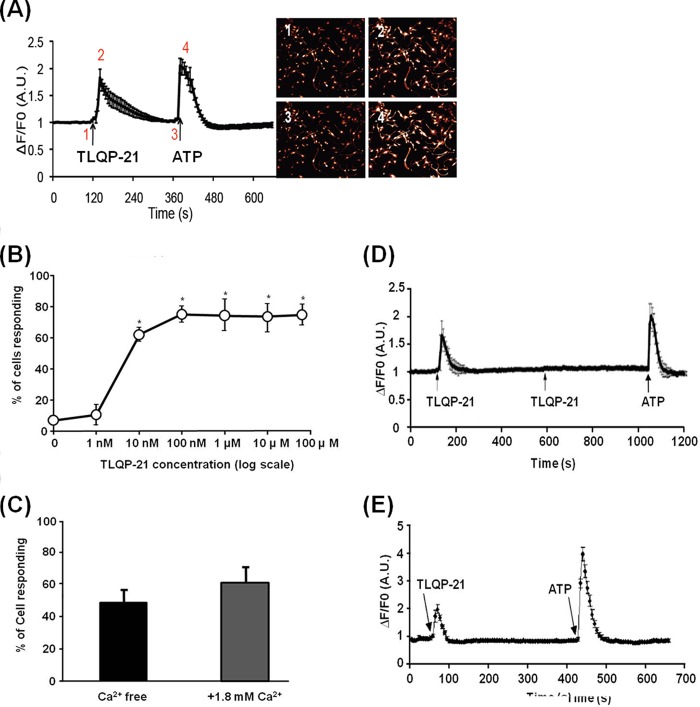
**TLQP-21 elicits intracellular Ca^2+^ increase in macrophages and microglia.** *A*, applying 100 nm TLQP-21 to cultured bone marrow-derived primary macrophages drives an increase in intracellular Ca^2+^. Cell images show the Fluo-4 fluorescence associated with different time points. The representative trace shows mean ± S.E. of cells (minimum of 78 cells were recorded for each experiments). *B*, dose response of TLQP-21 peptide in the Ca^2+^ assay used on macrophages is shown. *, *p* < 0.01, one-way ANOVA, Tukey's test, *n* = 4 (minimum of 78 cells were recorded for each experiment). *Error bars*, S.E. *C*, TLQP-21 elicited an increase in intracellular Ca^2+^ levels in extracellular Ca^2+^-free conditions (*p* = 0.173, *t* test, and one-way ANOVA). *D*, macrophages do not respond to the second 100 nm TLQP-21 application, although the ATP response was not affected. *E*, 100 nm TLQP-21 causes an increase in intracellular Ca^2+^ in cultured brain microglia. 100% of cells responded to 100 nm TLQP-21 (150 cells from a total of three biological replicates have been analyzed).

We tested whether rat microglia, macrophages, and DRG neurons respond to TLQP-21 by monitoring intracellular Ca^2+^ levels. 100 nm TLQP-21 caused a transient increase in intracellular Ca^2+^ levels in bone marrow-derived primary macrophages (Fig. 1*A*), and this Ca^2+^ increase was observed at TLQP-21 concentrations as low as 10 nm (Fig. 1*B*). ATP was used to test cell viability at the end of each experiment. This Ca^2+^ increase seems to be due to release of Ca^2+^ from their intracellular Ca^2+^ stores such as endoplasmic reticulum or mitochondria, because the Ca^2+^ increase was still observed when extracellular Ca^2+^ was depleted (Fig. 1*C*). Other VGF-derived peptides such as TLQP-62 and LQEQ-19 have also been implicated in pain processing (5, 6). We tested whether these VGF-derived peptides elicit increase in intracellular Ca^2+^ levels in cultured bone marrow-derived primary macrophages. Neither TLQP-62 nor LQEQ-19 caused an increase in intracellular Ca^2+^ levels in macrophages (data not shown). Interestingly, TLQP-21 induced desensitization to subsequent TLQP-21 treatments, but not to ATP treatments (Fig. 1*D*). This may indicate a biological desensitization occurring such as phosphorylation of the receptor. It is also possible that this could be due to depletion of Ca^2+^ in internal stores such as the endoplasmic reticulum; however, this is less likely because the desensitization occurs even with sufficient time (1 h) to recover and replenish the Ca^2+^, and ATP treatment gave an increase in intracellular Ca^2+^ levels. Actually, this desensitization by TLQP-21 has also been observed in cerebellar granule cells (17). These observations suggest that the effect of TLQP-21 on macrophages and microglia cells is a specific biological response and indicate the presence of a specific receptor for TLQP-21 in those cells. Brain-derived primary microglia also responded to TLQP-21 (Fig. 1*E*); however, 10 μm TLQP-21 did not induce changes in intracellular Ca^2+^ levels in cultured DRG neurons (data not shown).

##### Macrophages Stimulated with TLQP-21 Evoke Mechanical Hypersensitivity in Rats

**FIGURE 2.**
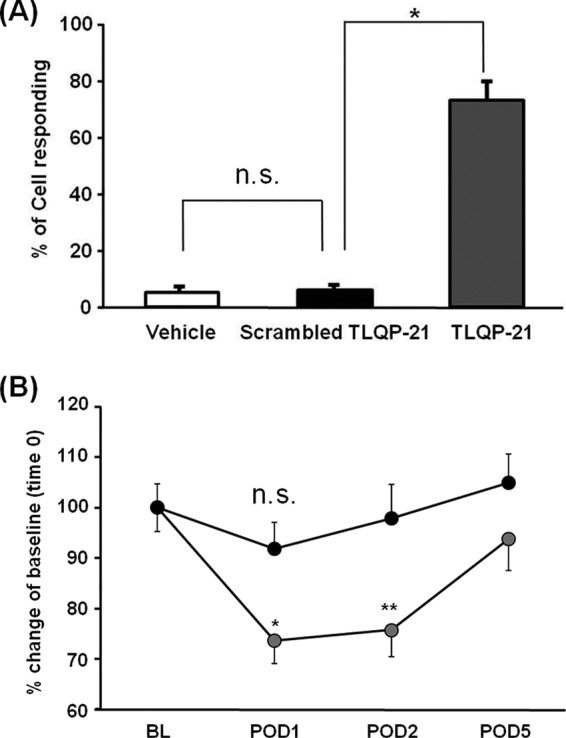
**Macrophages stimulated with TLQP-21 evoke mechanical hypersensitivity in rats.** *A*, scrambled TLQP-21 does not cause an increase in intracellular Ca^2+^ levels in macrophages (*p* = 0.995, one-way ANOVA, Tukey's test), whereas the percentage of cells responding to TLQP-21 was significantly increased (*, *p* < 0.001; *n.s*., not significant) compared with both vehicle and scrambled TLQP-21, *n* = 4 (78–154 cells for vehicle, 118–170 cells for scrambled TLQP-21, and 111–216 cells for TLQP-21 were analyzed). *B*, macrophages treated with TLQP-21 (*gray circles*) or ScrTLQP-21 (*black circles*) for 24 h were injected intraplantarly into rats. Injection of macrophages stimulated with TLQP-21 caused reduction of paw withdrawal thresholds at 24 h (*POD1*; *, *p* < 0.01 *versus* base line (*BL*)) and 48 h (*POD2*; **, *p* < 0.05 *versus* base line) after injection (*n* = 9, mean ± S.E. (*error bars*)). Two-way ANOVA Tukey's test, four or five rats were used for each experiment). Injection of macrophages incubated with ScrTLQP-21, however, did not affect the paw withdrawal threshold (*POD1*; *n.s.,* not significant, *p* = 0.67 *versus* base line). *n* = 9, mean ± S.E. Two-way ANOVA, Tukey's test. Four or five rats were used for each experiment.

A scrambled TLQP-21 (ScrTLQP-21) failed to induce an increase in intracellular Ca^2+^ levels in macrophages (Fig. 2*A*). This indicates that the phenomena observed in macrophages are specific to the sequence of TLQP-21 and are not due to charges of the peptide. We hypothesized that macrophages stimulated by TLQP-21 influence sensory neuronal excitability and alter mechanical sensitivity. We tested this hypothesis by measuring paw withdrawal thresholds in normal rats after intraplantar hind paw injection of cultured macrophages that had been pretreated with either TLQP-21 or ScrTLQP-21 for 24 h. Injection of ScrTLQP-21-treated macrophages did not affect the paw withdrawal threshold. In contrast, the paw withdrawal threshold decreased markedly 24 h after the injection of macrophages stimulated by TLQP-21, and this effect lasted for 48 h after injection (Fig. 2*B*).

##### gC1qR Was Identified as a Binding Protein for TLQP-21

**FIGURE 3.**
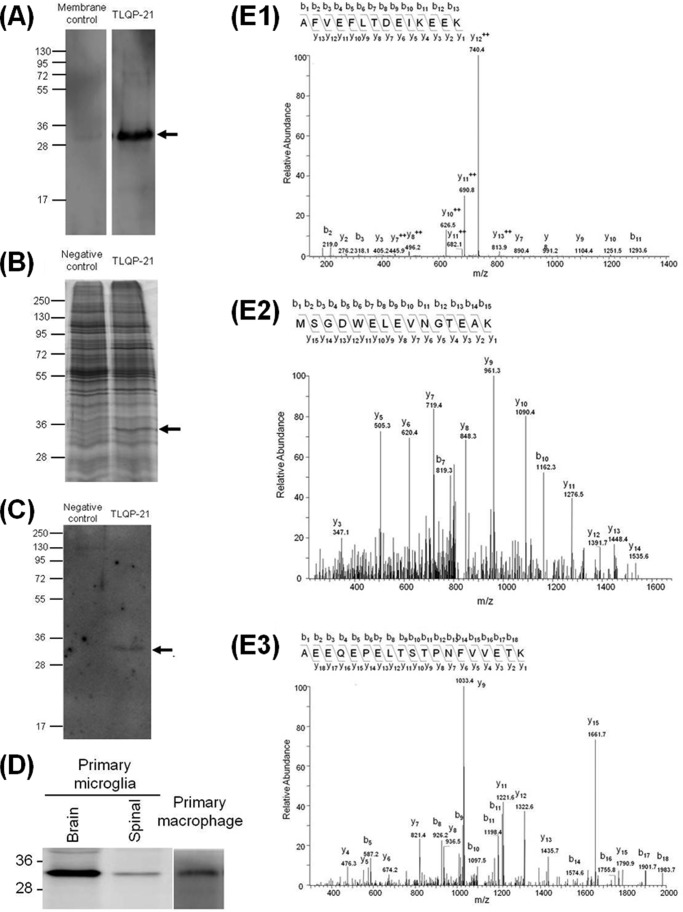
**gC1qR was identified as a binding protein for TLQP-21.** *A*, biotinylated TLQP-21 was conjugated to Sulfo-EMCS and applied to membrane fractions of rat brain. Samples were resolved using Tricine-PAGE and visualized by streptavidin-horseradish peroxidase. A clear band at ∼30 kDa (indicated by an *arrow*) was observed following cross-linking using biotinylated TLQP-21, but not in the membrane control lane using unconjugated cross-linker. *B*, membrane fractions of rat forebrain were applied to monomeric avidin column attached to TLQP-21. Proteins were eluted and resolved on SDS-PAGE followed by silver staining. A ∼30-kDa band (*arrow*) was apparent in the elutant from the TLQP-21-attached column, but not in monomeric avidin column only (*Negative control*). *C*, the gC1qR protein was eluted with TLQP-21 following monomeric avidin-based affinity chromatography. *D*, the gC1qR protein was expressed by both brain- and spinal cord-derived microglia and bone marrow-derived macrophages. *E*, the ∼30-kDa band was excised, proteins were in-gel digested with trypsin, and the resulting peptide mixture was analyzed by nano-LC-MS/MS. MS/MS spectra show three unique peptides (*E1–E3*) which have sequence identities to gC1qR.

To identify the receptor for TLQP-21, we used chemical cross-linking combined with mass spectrometry analysis. The modified TLQP-21 was used with a biotin covalently attached via the amide bond at the N terminus, and an extra cysteine residue was included at the C terminus. Sulfo-EMCS cross-linker was conjugated to the modified TLQP-21 via the sulfhydryl group of cysteine at the C terminus. This cross-linker-conjugated TLQP-21 was able to induce an increase in intracellular Ca^2+^ levels similar to the wild type TLQP-21 (data not shown). The cross-linking reaction was conducted by applying this modified peptide to membrane proteins from adult rat brains and spinal cord. Western blotting using streptavidin-HRP showed a ∼30-kDa band when the conjugated peptide was applied to the membrane proteins (Fig. 3*A*). This band was barely visible in the control consisting of the same amount of membrane protein with unconjugated cross-linker. We then attached TLQP-21 to monomeric avidin resin and incubated it with membrane proteins of forebrain tissue from postnatal day 4 rats. The potential receptor-TLQP-21 complex was eluted and compared with samples from a monomeric avidin-only column. The silver-stained SDS-polyacrylamide gels showed a ∼30-kDa band in the elution from TLQP-21 attached, but not in the column without TLQP-21 attached (Fig. 3*B*). To elucidate the identity of the ∼30-kDa protein, which was apparent in two different experiments, the ∼30-kDa band from the monomeric avidin experiment was excised followed by in-gel digestion of proteins with trypsin. The resulting peptide mixture was then analyzed by a nano-LC-MS/MS. We identified three unique peptides that have sequence identities to gC1qR (Fig. 3*E*). The complement-binding protein, gC1qR, is a highly acidic ∼30-kDa protein, ubiquitously expressed and binds to the globular heads of C1q, the first subcomponent of the classical pathway for complement activation. It has been observed that gC1qR is a multifunctional protein (18). Western blot analysis confirms that the gC1qR protein appears at ∼30 kDa in the elutions for the column with TLQP-21 attached, but not in the corresponding negative controls (Fig. 3*C*). Western blot analysis also shows that both macrophage and microglia cells express gC1qR (Fig. 3*D*).

##### The Activation of Macrophages by TLQP-21 Is Dependent upon gC1qR, and Blocking gC1qR Leads to Delayed Onset of Hypersensitivity Associated with PSNL

**FIGURE 4.**
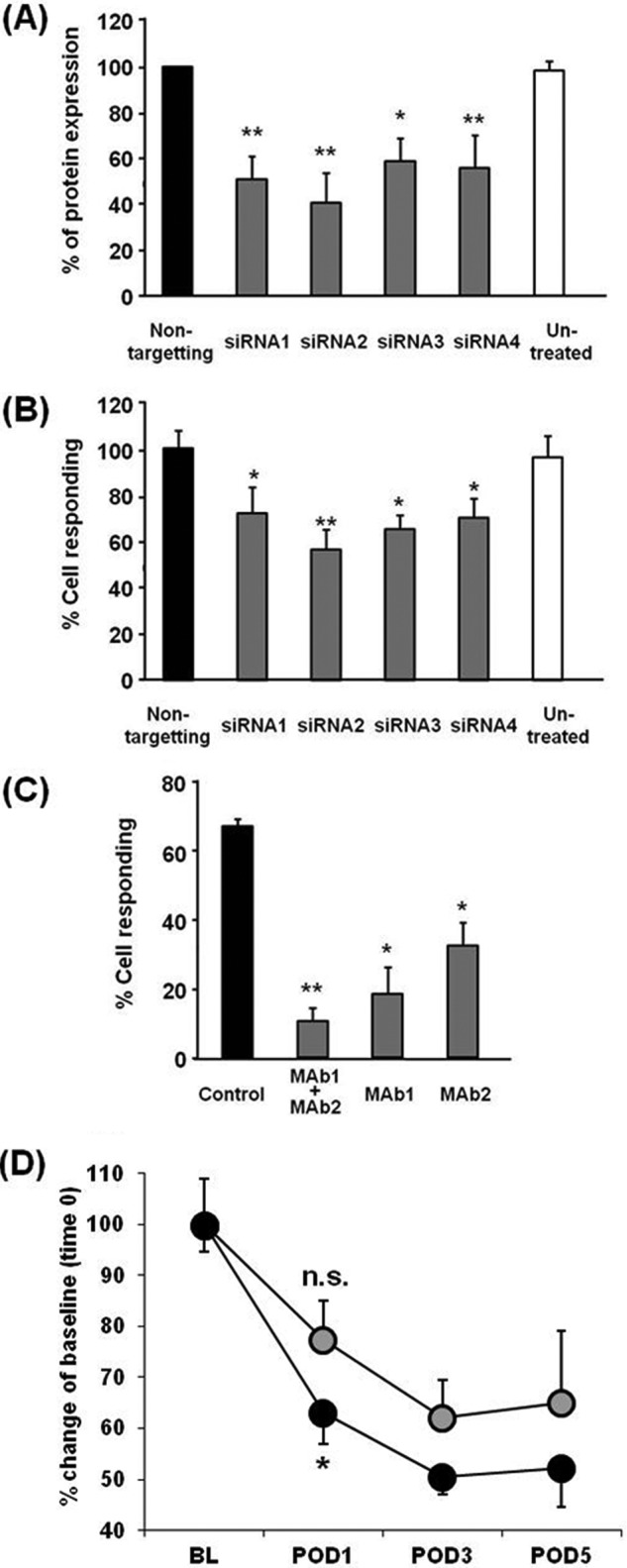
**Blocking gC1qR causes attenuation of the TLQP-21-induced increase in intracellular Ca^2+^ levels in macrophages and mildly reverses mechanical hypersensitivity in PSNL model.** *A*, transfection of siRNA against gC1qR (*siRNA 1–4*) into macrophages successfully reduced gC1qR protein expression. Nonspecific, negative control siRNA did not alter the gC1qR expression. *, *p* < 0.05; **, *p* < 0.01; one-way ANOVA, Fisher's test, *n* = 3. *B*, transfection of siRNA against gC1qR into macrophages caused significant reduction in the number of cells responding to TLQP-21. *, *p* < 0.05; **, *p* < 0.01; one-way ANOVA, Fisher's test, *n* = 4, a minimum of 183 cells were recorded for each experiment. *C*, preincubation (15 min) of macrophages with anti-gC1qR antibodies (MAb1, MAb2, 3 μg/ml each) significantly attenuates the TLQP-21-induced increase in intracellular Ca^2+^ levels. *, *p* < 0.01; **, *p* < 0.05; one-way ANOVA, Tukey's test, *n* = 3, a minimum of 80 cells were analyzed for each experiment. *D*, time course of paw withdrawal thresholds to von Frey mechanical stimulation on the left hind paw of PSNL model rats is shown. The left sciatic nerve was tied with silk thread and wrapped loosely with a cellulose membrane previously soaked in PBS containing either 25 μg of anti-gC1qR (MAb1, *gray circles*) or control antibody (*black circles*). The animals treated with control antibody showed a reduction of mechanical threshold 24 h after PSNL (*POD1*; *, *p* < 0.001 *versus* base line (*BL*)). The rats treated with gC1qR antibody, in contrast, showed a higher mechanical threshold compared with control antibody-treated rats, and their mechanical threshold 24 h after PSNL was statistically not different from the base line (*POD1*; *n.s*., not significant; *p* = 0.12 *versus* base line; ANOVA, Tukey's test, *n* = 9, 3, or 4 rats were used for each experiment).

To address whether the increase in intracellular Ca^2+^ levels in macrophages evoked by TLQP-21 depends on gC1Rq, we examined two approaches to silence gC1qR. First, we studied the effects of siRNA against gC1qR. Electroporation of four different siRNAs against gC1qR into macrophages significantly reduced the protein levels of gC1qR (Fig. 4*A*). These siRNAs successfully reduced the number of macrophage cells responding to TLQP-21 analyzed by live cell Ca^2+^ imaging (Fig. 4*B*). We then examined whether neutralizing gC1qR antibodies attenuate the intracellular Ca^2+^ levels in macrophages elicited by TLQP-21. Preincubation of macrophages for 15 min with neutralizing gC1qR monoclonal antibodies (MAb1 and MAb2) resulted in significant reduction of the response to TLQP-21 (Fig. 4*C*).

**FIGURE 5.**
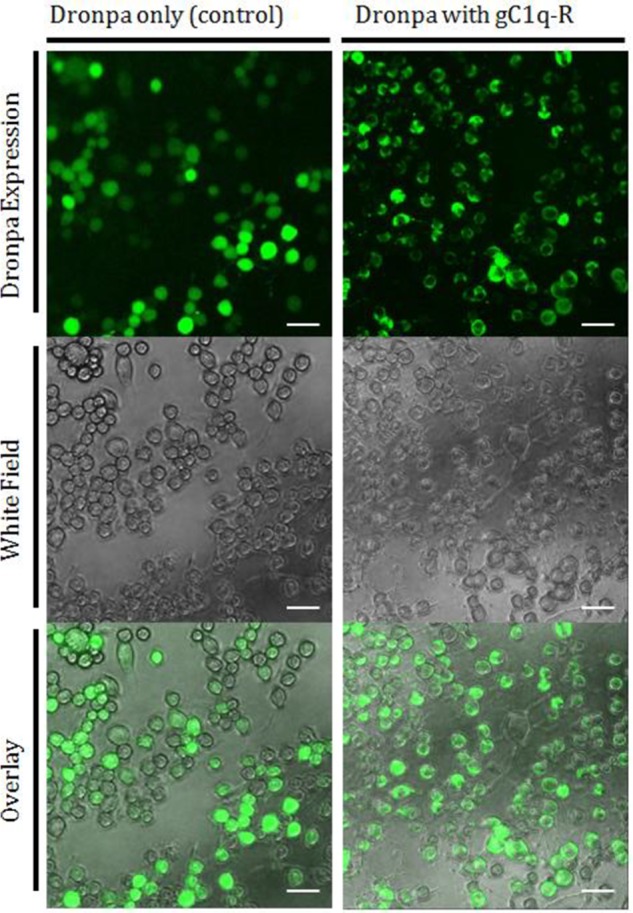
**Heterologously expressed gC1q-R in HEK 293 cells showed specific localization on the plasma membrane.** HEK 293 cells were transfected with pDG1-MN1 vectors (expressing green fluorescence protein Dronpa) or rat gC1q-R cDNA cloned into pDG1-MN1 vectors (expressing gC1q-R/Dronpa chimeric protein). The cells transfected with control pDG1-MN1 vectors express Dronpa all over them. However, most of the gC1q-R/Dronpa chimeric proteins were located specifically in the plasma membrane as green fluorescent rings can be observed. The *scale bars* are all 50 μm.

We then cloned rat gC1qR cDNA into pDG1-MN1 vector to create a chimera with green fluorescence Dronpa, and the construct was heterologously expressed in HEK 293 cells. The gC1qR-Dronpa fusion protein is found to be localized on the plasma membrane region as it shows a specific ring shape signal along the perimeter of the cells (Fig. 5). These transfected cells failed to respond to TLQP-21 in calcium imaging (data not shown). This may be due to lack of certain signaling molecules which link gC1qR and intracellular calcium release.

To examine the role of gC1qR in pain pathways, the gC1qR antibody (MAb1) was applied by a strip of oxidized cellulose membrane to the site of nerve ligation of PSNL model rats. After 24 h of PSNL, control IgG-treated rats showed a reduction of punctate mechanical threshold. In contrast, application of the gC1qR antibody delayed the onset of hypersensitivity associated with PSNL (Fig. 4*D*). These results suggest that macrophages stimulated by TLQP-21 via its receptor gC1qR initiate hyperexcitation of sensory neurons.

## DISCUSSION

gC1qR was originally identified as a protein with high affinity for the globular heads of the complement component C1q. gC1qR is a 33-kDa protein with a doughnut-shaped trimer structure and can form a disulfide bond between two gC1qR trimers, resulting in a hexameric structure. Fibroblasts expressing gC1qR induced a rapid and transient increase in intracellular Ca^2+^ levels via an IP_3_-dependent pathway upon application of C1q (19). Interestingly, the expression of gC1qR in adipocytes is induced during adipogenesis (20), and obese mice fed on a high fat diet showed increased density of TLQP-21 binding site in adipose tissues (10). Furthermore, gC1qR down-regulation in adipocytes prevented insulin-induced glucose uptake (20). These findings suggest that the TLQP-21/gC1qR pathway may also be important in adipogenesis. It has been observed that gC1qR can bind both high molecular weight kininogen (HK) and factor XII, and the HK-gC1qR interaction plays an important role in bradykinin generation in macrophages (21). Thus, gC1qR can bind plasma proteins such as C1q and HK, which in turn generates an inflammatory response from both the complement and kinin/kallikrein systems and initiates a plethora of biological responses. Recently Hannedouche *et al.* reported the complement receptor C3AR1 as a receptor for TLQP-21 (22). Although gC1qR and C3AR1 are known as receptors for complement protein C1q and C3a, respectively, it is not known whether they interact with each other. There is a possibility that TLQP-21 interacts with both gC1qR and C3AR1, either at the same time or sequentially, on the surface of macrophages to activate the cells. Such activation may lead to production and release of bioactive molecules such as cytokines. It would be interesting to see whether any particular cytokines are dysregulated in macrophages upon TLQP-21 stimulation and whether such cytokines may have direct effects on hypersensitivity of sensory neurons.

We have shown that TLQP-21 induces increase in intracellular Ca^2+^ levels in macrophages in a gC1qR-dependent manner. We also found that TLQP-21-stimulated macrophages activate sensory neurons either directly or indirectly and cause mechanical hypersensitivity in rats. The elevated intracellular Ca^2+^ concentration in macrophages leads to production of multiple molecules including nitric oxide and TNF-α (23, 24). It has also been shown that an induction of IL-8 expression occurs via gC1qR, and this is mediated through MAP kinase-dependent processes (25). It is thus also plausible that stimulation of macrophages (and microglia) with TLQP-21 may lead to production and secretion of some cytokines through gC1qR and possibly MAP kinase-dependent pathways. Such cytokines may be responsible for the activation of the sensory neurons. The involvement of macrophages in neuropathic pain pathogenesis has recently been highlighted (26). Resident macrophages in DRG proliferate after nerve injury (27), and circulating monocytes are recruited into the site of injury (28). Depletion of macrophages reduces mechanical hypersensitivity after nerve injury (29) and delays progression of neuropathic pain in diabetic model rats (30). We identified Pap/Reg2, a macrophage chemoattractant, as another molecule commonly up-regulated in the three disparate models of neuropathic pain (1). These findings indicate that macrophages stimulated by TLQP-21 through gC1qR may be involved in the development and/or maintenance of neuropathic pain, and disrupting TLQP-21/gC1qR interaction and/or its downstream signaling may provide a new way of controlling chronic pain.

The heterologously expressed gC1qR in HEK 293 cells failed to elicit intracellular Ca^2+^ increase upon TLQP-21 stimulation. This may be because HEK 293 cells do not express certain molecules required for gC1qR-dependent intracellular Ca^2+^ release. The C3AR1-transfected HEK 293 cells have been shown to respond to TLQP-21 after priming the cells with ATP (22). Although there is no evidence that gC1qR shares the same intracellular signaling pathway with C3AR1, priming the gC1qR-transfected HEK 293 cells with ATP may confer responsiveness to TLQP-21. We have shown that *in vivo* application of the neutralizing gC1qR antibody successfully reduced the mechanical hypersensitivity in neuropathic pain model rats. This effect, however, did not last >24 h. One plausible reason for this is degradation of the antibody, as we applied the antibody only once at the time of surgery. Continuous infusion of the antibody using an implantable osmotic pump may help enhancing the effect of the antibody, although precise positioning of the apparatus at the site of injury is challenging. Systemic injection makes it much easier to perform multiple application of the antibody; however, this method requires a large amount of the antibody.

TLQP-21 induces increase in intracellular Ca^2+^ levels in macrophages in a gC1qR-dependent manner. We also found that TLQP-21-stimulated macrophages activate sensory neurons either directly or indirectly and cause mechanical hypersensitivity in rats.

Interestingly, C1q has been shown to be up-regulated in the spinal cord of neuropathic pain model mice, and intrathecal application of C1q caused significant attenuation of persistent inflammation-induced mechanical and thermal hypersensitivity (31). This seems to contradict our findings. However, it is not known whether the effect of C1q in spinal cord is via binding to gC1qR. C1q has been shown to interact with multiple molecules such as cardiolipin (32), calreticulin (33), pentraxins (34), α2β1 integrin (35), and DNA (36) in addition to C1r and C1s complement proteins and antibody-antigen complexes. Most of the C1q-mediated biological responses are independent of gC1qR. However, C1q appears to be one of many ligands for gC1qR. In addition to HK and factor XII, gC1qR has been found to bind to diverse ligands including splicing factor ASF/SF2 (37), hyaluronan (38), and protein kinase Cμ (39). C1q is not particularly required for gC1qR to associate with these molecules. These findings indicate that the activation of macrophages by TLQP-21 through binding to gC1qR may not involve C1q.

Neuropeptide precursor VGF is commonly up-regulated in sensory neurons in a number of neuropathic pain models. A number of VGF-derived peptides have been identified, and some of them have been shown to play important roles in pain pathways. However, cell surface receptors for those VGF-derived peptides have not been identified. One of the VGF-derived peptides, TLQP-21, causes an increase in intracellular Ca^2+^ levels, which could lead to an activation of the cells. Inoculation of TLQP-21-stimulated macrophages into rat hind paw caused mechanical hypersensitivity. We identified gC1qR as a specific receptor for TLQP-21 in this study. gC1qR is a promising candidate receptor for TLQP-21, as blockade of qC1qR results in attenuation of the intracellular Ca^2+^ increase. This has also been confirmed by *in vivo* experiment, as application of the gC1qR-neutralizing antibody to the sciatic nerve of neuropathic pain model rats resulted in a delayed onset of mechanical hypersensitivity.

## Footnotes

4 The abbreviations used are:

DRG: dorsal root ganglia
ANOVA: analysis of variance
HK: high molecular weight kininogen
MS/MS: tandem mass spectrometry
Scr: scrambled
Tricine: *N*-tris(hydroxymethyl)methylglycine
PSNL: partial sciatic nerve ligation.
